# Reporting of biochemical blood values in COVID-19 patients: A retrospective study

**DOI:** 10.1097/MD.0000000000035833

**Published:** 2023-11-03

**Authors:** Berna Eriten, Semih Eriten

**Affiliations:** a Şehit Prof. Dr. İlhan Varank Sancaktepe Training and Research Hospital, Department of Pathology, Emek, İstanbul, Turkey; b Sultanbeyli State Hospital, Department of Emergency, Battalgazi, Sultanbeyli, İstanbul, Turkey.

**Keywords:** biochemical, biomarker, COVID-19, pandemic

## Abstract

**Background:**

This study aimed to investigate the impact of biochemical blood parameters on the progression of coronavirus disease (COVID-19). This retrospective study analyzed the clinical characteristics and biochemical test parameters of 352 COVID-19 patients treated at Malatya Training and Research Hospital in Turkey between March 3, 2021, and February 28, 2022.

**Methods:**

The methodological approach of our study included specific analyses and examinations to assess the effect of biochemical blood values on disease progression in COVID-19 patients. To achieve this aim, blood samples were collected from patients and various biochemical tests were performed. The data obtained were evaluated using statistical analyses to examine the relationship between these specific biochemical blood values and the severity of COVID-19.

**Results:**

High glucose, urea, alkaline phosphatase and lactate dehydrogenase levels and low albumin and potassium levels were associated with a more severe disease course. The results showed a significant link between certain biochemical blood values and the severity of COVID-19. These findings suggest that these markers may serve as valuable clinical indicators for predicting the disease progression and severity.

**Conclusion:**

This study demonstrates the importance of monitoring and analyzing biochemical blood values as essential tools for assessing the severity and progression of COVID-19. The identified markers provide valuable information regarding the prognosis of the disease and may help healthcare professionals make informed decisions regarding patient care.

## 1. Introduction

The global epidemic, known as coronavirus disease (COVID-19), was first observed in 2019 in Wuhan, China.^[1]^ This recently discovered epidemic continues to spread worldwide as a severe and contagious disease.^[2]^ COVID-19 has a variable clinical presentation, ranging from asymptomatic to severe illness, leading to death.^[3]^

Studies to develop effective treatments for COVID-19 are ongoing.^[4–10]^ Although many new treatment options are available to combat COVID-19, the success rates vary. Therefore, laboratory tests play an important role in predicting the severity and course of the disease and in determining the appropriate treatment.^[11]^

Although studies in the literature have broadly defined the clinical features of COVID-19, the changes in the most common biochemical parameters reported in patients with COVID-19 are still not fully determined.^[2]^ At the same time, it is seen that there are certain differences in the results due to the different designs of the studies and insufficient sample sizes.^[11]^

In a recent study conducted in Turkey, the presence of cardiovascular disease, chronic renal failure, an increase in the number of malignancies and comorbidities, complaints of loss of appetite, lack of fever, decrease in oxygen saturation value, increased pulse rate, maximum D-dimer, aspartate aminotransferase, urea, creatinine values, troponin, lactate dehydrogenase (LDH), maximum LDH, ferritin, and maximum ferritin; Factors such as C-reactive protein (CRP), maximum CRP, procalcitonin, maximum procalcitonin, potassium values, and low albumin values have been associated with bacterial infection, heart disease, acute respiratory complications distress syndrome, failure of liver function tests, arrhythmia, and shock conditions. Therefore, the need for corticosteroid and pulse corticosteroid treatment has been shown to increase mortality.^[12]^

Since biochemical changes in the blood play an important role in predicting the condition and prognosis of patients, guiding treatment, and even evaluating the curative effect, there is a need to obtain more convincing biochemical results.^[11–15]^ In this study, we aimed to evaluate the changes in the biochemical parameters of COVID-19 patients according to their clinical characteristics.

## 2. Method

### 2.1. Data collecting

First, this was a retrospective study. For the treatment of COVID-19, the clinical features and biochemical test parameters of 243 patients who were treated at Malatya Training and Research Hospital in Malatya province of Turkey between March 3, 2021, and February 28, 2022, were used. Data were obtained from the Malatya Training and Research Hospital Patient Registration Automation Program. The study was approved by the Ethics Review Board of the Malatya Training and Research Hospital (Approval Number: 23536505-000-13874). Because this was a retrospective study, informed consent was not obtained. The patients were not actively involved in any new interventions or interactions. Only existing data and medical records were analyzed. Since the study was based on preexisting data only and did not require the direct involvement of patients, informed consent was not required.

The biochemical blood tests used in the study were performed using Roche Cobas 6000. Our average reference ranges accepted for healthy individuals are as follows: Glucose: 70–105 mg/dL, Urea:15–45 mg/dL, aspartate aminotransferase (AST): 0 to 50 U/L, alkaline phosphatase (ALP): 40 to 150 U/L, gamma-glutamyl transferase: 12 to 64 U/L, LDH: 90 to 250 IU/L, creatine kinase (CK): 30 to 200 U/L, creatine kinase-MB: 0 to 25 U/L, total bilirubin (T. bilirubin): 0.2 to 1.2 mg/dL, direct bilirubin: 0 to 0.5 mg/dL, Protein: 6.4 to 8.5 g/dL, Albumin: 3.5 to 5.5 g/dL, Calcium: 8.5 to 10.6 mg/dL, Phosphorus: 2.5 to 4.5 mg/dL, CRP: 0 to 5 mg/L, Potassium: 3.5 to 5.2 mmol/L, Chlorine: 85 to 120 mEq/L, Sodium: 136 to 148 mEq/L.

Patients with previous hospital records and biochemical blood values within the normal reference ranges and patients with positive PCR test results and biochemical blood values outside the normal reference ranges were included in our study.

### 2.2. Statistical analysis

In this study, descriptive statistics and categorical variables are shown as numbers and percentages, and continuous variables as medians (minimum-maximum). The chi-squared test was used to examine the relationship between the 2 independent categorical variables. The Mann–Whitney *U* test was used to test the significance of the difference between the means of the 2 non-parametric variables. The diagnostic test performance of biochemical blood parameters was tested using ROC analysis. Multivariate logistic regression analysis was performed to evaluate statistically significant independent factors. Statistical analyses were performed using “IBM SPSS Statistics (version 25.0) for Windows and Jamovi (version 2.3) for Windows software.”

## 3. Findings

In this study, there was a significant relationship between the discharge/ex status of the patients and their age ($X^{2}=29.39$, P < .05), sex ($X^{2}\;=6.74$, *P* < .05), chronic disease ($X^{2}=21.72$, *P* < .05), and duration of hospitalization ($X^{2}\;=23.41$, *P* < .05) (Table 1).

**Table 1:** Comparison of Ex and discharge status of patients according to demographic variables.

Categories	Discharge	Ex	$X^{2}$	*P*
Age				
<65	130 (87.8%)	18 (12.2%)	29.39	.00*
≥65	126 (61.8%)	78 (38.2%)		
Gender				
Female	107 (66.0%)	55 (34%)	6.74	.009*
Male	149 (78.4%)	41 (21.6%)		
Chronic disease				
Yes	64 (56.6%)	49 (43.4%)	21.72	.00*
No	192 (80.3%)	47 (19.7%)		
Duration of hospitalization				
<7	105 (79.5%)	27 (20.5%)	23.41	.014*
≥7	142 (67.3%)	69 (32.7%)		

* *P* < .05 significant difference, Chi Square.

The significance of the difference between the exit and discharge was tested using the Mann–Whitney *U* test. Statistically significant differences were found between exit and discharge. According to the results, glucose, urea, AST, ALP, LDH, CK, bilirubin, bilirubin, sodium, potassium, and CRP values, mean age, and length of hospital stay were higher in ex-patients. Protein, albumin, and calcium levels were lower (Table 2).

**Table 2:** Comparison of measurements by discharge and Ex condition.

	Discharge	Ex	U	*P*
	Mean (Min-Max)	Mean (Min-Max)		
Age	63.36 (21–94)	75.93 (55–93)	6494	.00*
Duration of hospitalization	8.63 (0–58)	15.16 (1–65)	8268	.00*
Glucose	145.32 (28–547)	198.45 (16–476)	7109	.00*
Urea	46.53 (1–338)	121.76 (11–405)	3861.5	.00*
AST	38.5 (9–913)	149.25 (11–2898)	9132	.00*
ALP	90.92 (32–452)	140.77 (35–514)	7944	.00*
GGT	66.13 (5–408)	87.16 (12–669)	10727.5	.14
LDH	326.06 (26–1995)	701.91 (128–4151)	3698.5	.00*
CK	110 (14–1742)	271.71 (23–4002)	7212.5	.00*
CK-MB	14.97 (0–88)	21.3 (1–103)	5881.5	.09
T. bilirubin	0.66 (0–16)	1.54 (0–15)	6480	.00*
D. bilirubin	0.2 (0–9)	0.75 (0–9)	4902.5	.00*
Protein	6.37 (4–8)	5.49 (3–7)	5623	.00*
Albumin	3.3 (2–5)	2.47 (1–4)	3952.5	.00*
Calcium	8.09 (3–11)	6.71 (2–15)	7185	.00*
Phosphorus	3.12 (1–10)	3.26 (2–16)	6812.5	.57
Sodium	136.07 (127–188)	136.86 (3–164)	9919	.03*
Potassium	10.62 (3–515)	4.9 (2–8)	10017.5	.02*
Chlorine	103.61 (91–148)	104.14 (94–121)	7991.5	.78
CRP	5.73 (0–247)	12.45 (0–38)	4608.5	.00*

ALP = alkaline phosphatase, AST = aspartate aminotransferase, CK = creatine kinase, CK-MB = creatine kinase-MB, CRP = C-reactive protein, D. bilirubin = direct bilirubin, GGT = gamma-glutamyl transferase, LDH = lactate dehydrogenase, T. bilirubin = total bilirubin.

* *P* < .05 significant difference. Mann-Whitney *U*.

**Figure 1.**
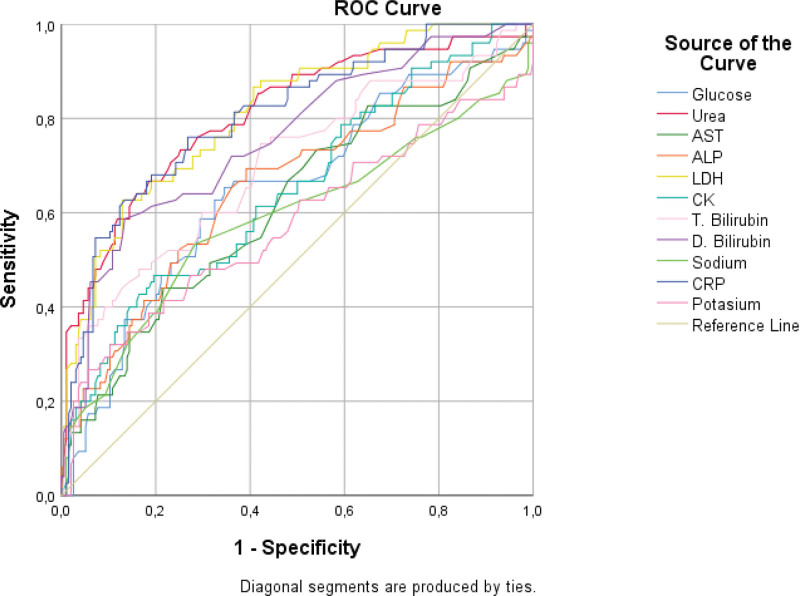
Sensitivity and specificity curve according to mean values (increasing value for Ex).

**Figure 2.**
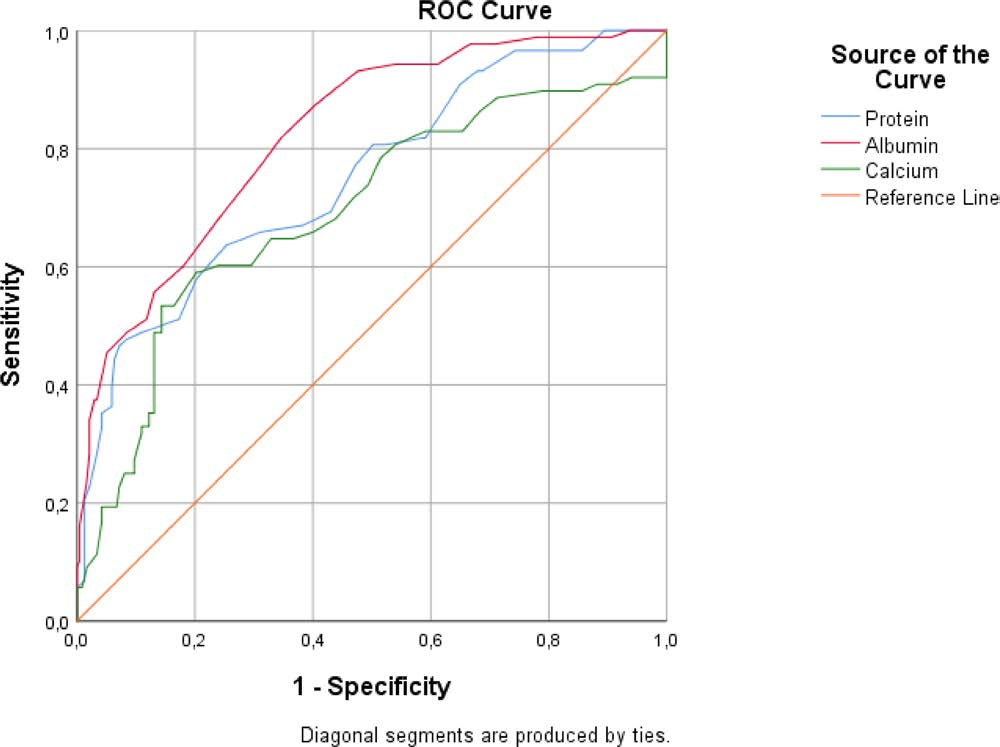
Sensitivity and specificity curve according to mean values (decreasing value for Ex).

The COVID-19 diagnostic test performances of the biochemical blood values that were significant in the comparison analyses of the discharge and Ex groups were tested using ROC analysis. In the literature, an area under the curve (AUC) of 0.5 suggests no discrimination (i.e., ability to diagnose patients with and without the disease or condition based on the test), 0.7 to 0.8 is considered acceptable, 0.8 to 0.9 is considered excellent, and more than 0.9 is considered outstanding.^[16,17]^ According to the results obtained, when the area under the ROC Curve (AUC) values were analyzed, the values for urea (AUC = 0.861), LDH (AUC = 0.864), albumin (AUC = 0.863), bilirubin (AUC = 0.818), and CRP (AUC = 0.861) were excellent. The AUC values indicated that the classification ability of the model was high. In particular, the highest AUC value obtained for Ex (mortality) prediction was for LDH (AUC = 0.864). (Table 3). In our study, the diagnostic performance was evaluated for each blood value, and ROC curves were drawn by determining the cutoff points (Figs. 1 and 2).

**Table 3:** Diagnostic performance of biochemical blood parameters.

n = 352	AUC (95% CI)	*P*	Cutoff	Sensitivity (%)	Specificity (%)	PPV (%)	NPV (%)
Glucose	0.710 (0.57–0.72)	.00	≥129	76%	61.51%	35.85%	90.06%
Urea	0.861 (0.74–0.87)	.00	≥52	83.95%	71.94%	48.92%	93.33%
AST	0.669 (0.54–0.69)	.00	≥40	53.01%	74.53%	39.29%	83.61%
ALP	0.661 (0.58–0.73)	.00	≥82	72.84%	56.82%	34.1%	87.21%
LDH	0.864 (0.75–0.86)	.00	≥344	86.25%	69.83%	48.59%	93.89%
CK	0.706 (0.57–0.72)	.00	≥108.98	53.09%	75.73%	42.57%	82.65%
T. bilirubin	0.725 (0.62–0.77)	.00	≥0.71	60%	77.52%	45.28%	86.21%
D. bilirubin	0.818 (0.69–0.82)	.00	≥0.2	73.17%	81.18%	55.56%	90.39%
Protein	0.770 (0.69–0.81)	.00	≥5.2	47.50%	93.31%	69.09%	84.95%
Albumin	0.77 (0.80–0.87)	.00	≥2.6	65.85%	86.59%	60.67%	88.98%
Calcium	0.728 (0.62–0.76)	.00	≥6.7	53.75%	88.30%	58.11%	86.35%
Sodium	0.571 (0.50–0.67)	.02	≥140	45%	72.35%	33.03%	81.28%
Potassium	0.602 (0.49–0.66)	.04	≥4.84	53.85	73.82	35.9	85.45
CRP	0.861 (0.74–0.87)	.00	≥3.77	87.8%	68.82%	46.75%	94.76%

ALP = alkaline phosphatase, AST = aspartate aminotransferase, CK = creatine kinase, CRP = C-reactive protein, D. bilirubin = direct bilirubin, LDH = lactate dehydrogenase, NPV: = “negative predictive value”, PPV = “positive predictive value”, T. bilirubin = total bilirubin.

Biochemical blood parameters that were found to be significant as a result of univariate analyses were included in the multivariate logistic regression model. First, a variance inflation factor (VIF) analysis was performed to detect multiple linear correlations. The VIF is calculated to determine the degree of relationship between an independent variable and other independent variables.^[18]^ If the VIF is greater than or equal to 10, there is a multicollinearity problem.^[19–21]^ In this study, the VIF values of T. bilirubin and D. bilirubin parameters were found to be above 10. T. bilirubin was removed from the model and the model was reestablished. The results of the established multivariate logistic regression analysis model showed that mean increases in glucose (OR:1.004, *P* < .05), urea (OR:1.019, *P* < .05), ALP (OR:1. 006, *P* < .05), LDH (OR:1.004, *P* < .05), albumin (OR:0.252, *P* < .05), and potassium (OR:0.507, *P* < .05) levels were effective in reducing the Ex (mortality) risk of patients. Other variables had no significant effect on mortality risk (Table 4).

**Table 4:** Evaluation of risk factors affecting Ex with multivariate logistic regression analysis.

	B	Sig.	Exp (B)	95% C.I. for EXP (B)	
				Lower	Upper
Glucose	0.004	0.03*	1.004	1	1.008
Urea	0.019	0.00*	1.019	1.008	1.031
AST	−0.002	0.08	0.998	0.995	1
ALP	0.006	0.01*	1.006	1.001	1.01
LDH	0.004	0.00*	1.004	1.002	1.005
CK	0.000	0.88	1.000	0.998	1.002
D. bilirubin	−0.053	0.75	0.948	0.679	1.323
Protein	0.167	0.64	1.182	0.589	2.374
Albumin	−1.38	0.01*	0.252	0.085	0.742
Calcium	−0.1	0.24	0.905	0.767	1.067
Sodium	−0.077	0.07	0.926	0.851	1.007
Potassium	−0.679	0.03*	0.507	0.28	0.919
CRP	−0.005	0.57	0.995	0.979	1.011

ALP = alkaline phosphatase, AST = aspartate aminotransferase, CK = creatine kinase, CRP = C-reactive protein, D. bilirubin = direct bilirubin, LDH = lactate dehydrogenase.

* *P* < .05 significant difference.

## 4. Discussion

In this study, age, chronic illness, length of hospital stay, and biochemical blood parameters of the patients were evaluated separately, and their effects on the risk of death were investigated. In our study, the mortality rate of patients aged ≥65 years was higher. Similarly, Ergenc et al^[22]^ found that patients with ≥ 65 deaths had higher mortality rates. Teker et al^[23]^ found that the course of COVID-19 worsened with increasing age and number of deaths. Unver-Ulusoy et al^[24]^ found that 68.8% of patients hospitalized in the intensive care unit were aged 65 years and older. According to information reported by the US Centers for Disease Control and Prevention, the mortality rate observed in ≥65 patients is higher^[25]^ and it is thought that weakened immune functions in elderly patients predispose to infection.^[26]^ Therefore, the mortality rate may be higher in patients aged >65 years.

In our study, the mortality rate was higher in patients with chronic diseases. Wang et al^[27]^ reported that elderly people are at a higher risk for chronic diseases and infections, and that mortality due to COVID-19 increases in those with hypertension and coronary heart disease. Reyes-Sánchez et al^[28]^ In a study of adults who tested positive for COVID-19 in Mexico, Reyes-Sánchez et al^[28]^ revealed that chronic diseases may be associated with the severity of COVID-19 and deaths. Considering the information in the literature, the state of chronic illness can be used as a leading parameter in estimating the course of the disease.

In our study, we concluded that the risk of death from COVID-19 differed according to sex. Women had a higher risk of death than men. In the literature, the effect of gender on COVID-19 and mortality risk was investigated by Doerre and Doblhammer^[29]^ In the study, it was determined that the mortality rate in females were 2 times higher than in males. Ünver-Ulusoy et al^[24]^ found that men had a higher risk of death. Peckham et al^[30]^ found that male patients were almost 3 times more likely to be admitted to the intensive care unit and had a higher mortality rate than female patients. When the literature is analyzed, gender plays an important role in COVID-19-related deaths.

In our study, the duration of hospital stay was higher in patients who died. According to Rozman et al^[31]^ the probability of discharge within the first 7 days after hospitalization was 36.5%, while the probability of dying in the same period was 7.9%. On the 21st day, the probability of discharge increased to 72.9%, while the probability of death increased to 14.4%. Da Costa Sousa et al^[32]^ revealed that longer hospital stay may be related to age. More comprehensive studies are needed to explain the relationship between length of stay and death.

In our study, we investigated the diagnostic power of biochemical blood parameters of COVID-19 patients with investigated. Among the biochemical parameters included in the study, the AUC values of urea, LDH, albumin, bilirubin, and CRP were found to be high (0.8–0.9). Among these parameters, the highest AUC value was observed for CRP level. Logistic regression analysis was performed to determine the effects of biochemical parameters on the risk of death. The increase in Glucose, Urea, ALP and LDH levels increased the risk of death, and the increase in Albumin, Calcium and Potassium levels decreased the risk of death. In the study by Ergenç et al,^[22]^ white blood cell, procalcitonin, troponin, ferritin, monocyte, International Normalized Ratio, LDH, AST, ALT, mean corpuscular volume, and platelet values were found to be higher in the old ex. Ferrari et al^[33]^ stated that simple hematological tests can be used for the diagnosis of COVID-19 in developing countries where RT-PCR testing is limited.

### 4.1. Limits of the study

This study was conducted using data from a specific time period and geographical area. It should be noted that the generalizability of the results is limited. In addition, the retrospective design of the study may have made it difficult to determine cause-and-effect relationships. Finally, the data sources and measurement methods used in the study were limited to a specific institution and protocol, which limits the generalizability of the results to other health care institutions.

## 5. Conclusion

High Glucose, Urea, ALP and LDH levels and low Albumin and Potassium levels were associated with a more severe course of COVID-19. These results suggest a significant association between certain biochemical parameters and COVID-19 severity.

## Author contributions

**Conceptualization:** Semih Eriten.

**Data curation:** Berna Eriten.

**Formal analysis:** Berna Eriten.

**Methodology:** Berna Eriten.

**Project administration:** Semih Eriten.

**Resources:** Berna Eriten.

**Software:** Berna Eriten.

**Supervision:** Semih Eriten.

**Writing – original draft:** Berna Eriten.

**Writing – review & editing:** Berna Eriten.
